# Supplementary material for: TNBC Therapeutics Based on Combination of Fusarochromanone with EGFR Inhibitors
Source: Biomedicines. 2022 Nov 12;10(11):2906. doi: 10.3390/biomedicines10112906 (PMC9687139; doi:10.3390/biomedicines10112906)

Supplementary Materials

Figure S1: Crystal Violet Assay: Dose-dependent growth inhibition effect of FC101 in (MDA-MB231)

Figure S2: Western Blotting Experiments: Dose-dependent proteomics effect of FC101 in (MDA-MB231)

Figure S1. FC101 significantly inhibits proliferation of cultured TNBC cells (MDA-MB-231)

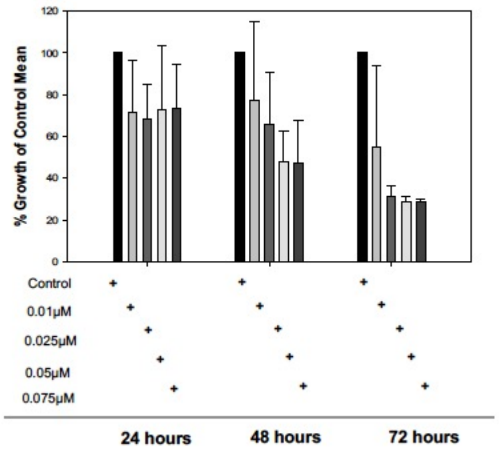

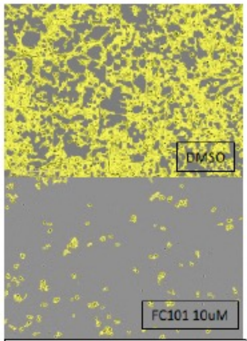

Figure 2: Phase contrast Images of TNBC Cells (MDA-MB231) treated with FC101 and DMSO, 120h post treatment.

Crystal Violet Assay

FC101 significantly inhibits cell growth in TNBC. TNBC cells at a density of 2 × 104 cells/well were seeded in a 96-well cell culture plate, treated with FC101 (0.01-0.075 µM) for 24, 48, and 72 h.

Figure S2. FC101 affects proteins in the dual oncogenic mTOR and MAPK signaling pathways downstream of EGFR (MDA-MB-231)- 24 h post-treatment

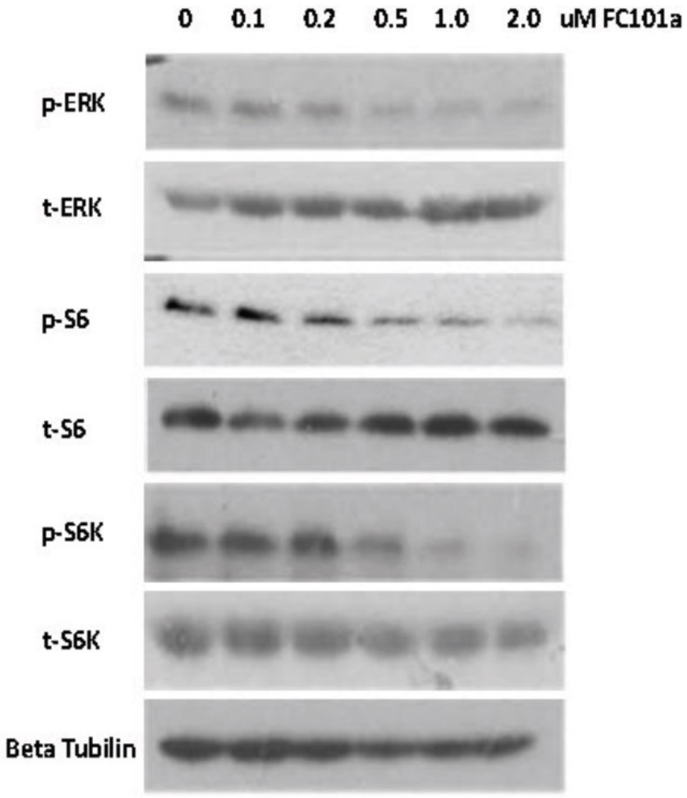

Supplement: Supplementary file 1 [file biomedicines-10-02906-s001.zip › biomedicines-1977898-supplementary.pdf]
